# A Case–Referent Study of Lung Cancer and Incense Smoke, Smoking, and Residential Radon in Chinese Men

**DOI:** 10.1289/ehp.1002790

**Published:** 2011-11-01

**Authors:** Lap Ah Tse, Ignatius Tak-sun Yu, Hong Qiu, Joseph Siu Kai Au, Xiao-rong Wang

**Affiliations:** 1 School of Public Health and Primary Care, The Chinese University of Hong Kong, Hong Kong Special Administrative Region, China; 2 Department of Clinical Oncology, Queen Elizabeth Hospital, Kowloon, Hong Kong Special Administrative Region, China

**Keywords:** incense burning, lung neoplasm, residential radon, smoking

## Abstract

Background: Burning incense generates large amounts of air pollutants, many of which are confirmed or suspected human lung carcinogens.

Objectives: We conducted a population-based case–referent study to examine the effect of incense smoke exposure on lung cancer risk among Chinese males and explored the joint effect of cigarette smoking and exposure to residential radon.

Methods: We recruited 1,208 male lung cancer incident cases and 1,069 community referents from 2004 to 2006 and estimated their lifetime exposures to incense smoke and other residential indoor air pollutants based on self-reported information collected during interviews. We performed unconditional multivariable logistic regression analysis to estimate the odds ratio (OR) for lung cancer associated with exposure to incense smoke after adjusting for possible confounders. We conducted stratified analyses by smoking status and exposures to incense burning and residential radon and explored the potential additive-scale interactions.

Results: We observed an association between incense exposure and lung cancer that was limited primarily to smokers. Cigarette smoking and high cumulative incense exposure at home appeared to have a synergistic effect on lung cancer (compared with never-smokers who never used incense, the OR for lung cancer for smokers who used incense ≥ 60 day-years = 5.00; 95% confidence interval: 3.34, 7.51). Power was limited, but we also found preliminary evidence suggesting that radon exposure may increase risk among smokers using incense.

Conclusion: Our study suggests that exposure to incense smoke in the home may increase the risk of lung cancer among smokers and that exposure to radon may further increase risk.

Indoor air pollution resulting from burning incense is a major public health problem in Hong Kong and other Asian countries, where burning incense to worship gods and ancestors is a popular ritual with a long traditional practice (Ho and Yu 2002; Lung et al. 2003). A previous survey showed that about half of Chinese families in Hong Kong burned incense at home, usually twice a day (Koo and Ho 1996). Burning incense generates large amounts of particulate matter (PM), aerosols, nitrogen dioxide, sulfur dioxide, formaldehyde, benzene, polycyclic aromatic hydrocarbons (PAHs), and other volatile organic compounds (Lin et al. 2008; Lu et al. 2008; Wang et al. 2007); many of these are confirmed or suspected human carcinogens associated with lung cancer [International Agency for Research on Cancer (IARC) 2010a, 2010b; Lin et al. 2008]. However, the association between burning incense and lung cancer is uncertain, and epidemiological evidence is very limited.

Previous studies of incense smoke exposure and lung cancer, which were based mainly on females, have not demonstrated a consistent association (Chan-Yeung et al. 2003; Chen et al. 1990; Ger et al. 1993; Koo and Ho 1996; MacLennan et al. 1977). Because incense smoke condensates contain hazardous agents similar to those found in tobacco smoke, including many that were mutagenic and genotoxic in the Ames test (Chen and Lee 1996), it is logical to hypothesize that exposure to incense smoke could also increase lung cancer risk. On the other hand, carcinogenic exposures may be much less intensive than those associated with active cigarette smoking, and an independent effect of incense burning on lung cancer might not be discernable in the general population.

Using data from a large population-based case–referent study conducted among Chinese males, we examined the association between incense smoke exposure and lung cancer, stratified by smoking status. We further explored the possible joint effect of incense smoke and exposure to residential radon.

## Materials and Methods

*Study design and the subjects.* The study design of this population-based case–referent study has been described previously, where an exposure–response relation between secondhand smoke (SHS) exposure and adenocarcinoma of the lung among never-smokers was observed (Tse et al. 2009). In brief, we consecutively interviewed 1,208 Chinese males 35–79 years of age with incident cases of primary histologically confirmed lung cancer from 1 February 2004 to 30 September 2006 at the largest oncology center in Hong Kong; the response rate was 96%. A study questionnaire about work, lifestyle, and health among Hong Kong males that was developed by the authors was completed by 1,148 cases (95%) and by 60 proxy respondents for those patients who were too ill to speak. Of the proxies, 90% were the wives of the patients (the other 10% were other household members), and the information they provided was subsequently confirmed by the patients. We further interviewed 1,069 male referents that were randomly selected from residential telephone directories for the same districts in which the cases resided, with a response rate of 48%. Each community referent was frequency matched to cases in 5-year age groups and had no history of physician-diagnosed cancer in any site. This study was approved by the ethics committees of both the Chinese University of Hong Kong and Queen Elizabeth Hospital. We complied with all applicable requirements of international regulations (including internal review board approval), and the participants gave written informed consent before the study.

*Data collection.* Personal interviews were carried out by trained interviewers to collect information on each subject, including demographic data, habits of tobacco smoking and alcohol drinking, dietary habits, cancer history in first-degree relatives, living density, ventilation and sources of indoor air pollution in the residence, and exposures to confirmed or suspected occupational carcinogens. Relevant medical information including medical diagnosis and histology was retrieved from the hospital records. Men were classified as ever-smokers if they had smoked > 20 packs of cigarettes or 12 oz of tobacco in their lifetime or smoked > 1 cigarette/day or > 1 cigar/week for 1 year (Ferris 1978). Exposure to confirmed or suspected occupational carcinogens was defined as ever regularly exposed (i.e., at least once a week for at least 6 months) to any of these agents: silica, asbestos, arsenic, nickel, chromium, tars, asphalts, painting, pesticide, diesel, cooking fume, and welding fume in the workplace (Tse et al. 2009).

*Assessment of indoor air pollutants.* We collected information on lifetime exposure to various indoor air pollutants in the home for each participant since childhood, including incense burning, residential radon exposure, SHS, years of cooking by frying, type of fuel use, and exposure to mosquito coil burning inside the house during summer months (never and ever).

Questions on SHS exposure in the home included “Did any person (including parents, spouse, children, or any other relatives or friends) who had been living with you (since your childhood) smoke any tobacco product regularly in your presence at home? If so, what is the relationship between you and the smoker, and how long has he (or she) smoked?” We also collected information on SHS exposure of participants from each workplace by asking the question “Did any person who had been working with you smoke any tobacco product regularly in your presence?” SHS exposure was defined as ever lived or worked with a smoker for at least 1 year and was regularly exposed to tobacco smoke (Tse et al. 2009; Yu et al. 2006a).

We collected information on incense smoke exposure at home using these questions: “Did any person including yourself burn any incense products (e.g., joss sticks, scented coil) inside your home? If yes, how often did such person burn incense products inside the room (i.e., the frequency: times a day, times a week, times a month), and how many years has such habit been maintained?” Ever exposure to incense smoke referred to inhalation of incense smoke at least once per month for a year or more. Lifetime cumulative exposure to incense smoke in the home was the product of frequency of exposure to incense smoke per day by years of exposure. Cumulative residential radon exposure was assessed semiquantitatively based on detailed information about all residences during their lifetime, including building age, floor level, window-opening practices, building materials, and wall surface–covering materials according to an established formula recommended by Hong Kong Government (Lee 1997). This formula was derived from a territory-wide indoor radon survey in Hong Kong, as described in detail by Yu et al. (2006a) in their Appendix A. The score of cumulative residential radon exposure does not have a unit, and a higher score indicates a higher level of exposure to residential radon.

*Statistical analysis.* Chi-square tests or *t*-tests were used to examine differences of incense smoke exposure and other selected risk factors between the cases and referents. We used the median of exposed community referents as the cut point to classify low and high exposure levels for the frequency of incense burning (1–2 times/day and ≥ 2 times/day) and cumulative exposure (1–60 day-years and ≥ 60 day-years, where 2 day-years is equivalent to burning incense twice a day for 1 year, or once a day for 2 years, etc.), respectively, whereas those never exposed to incense burning were treated separately as the reference group. Cumulative residential radon exposure was classified according to the first, second, third, and fourth quartiles (< 7.78, 7.78 to < 8.86, ≥ 8.86 to < 9.62, ≥ 9.62, respectively), based on the distribution of exposure scores in referents, and to low (score < 8.86) and high (score ≥ 8.86) levels, based on the median score in referents. A radon-missing category was created, and it was adjusted for in the model. We fitted unconditional multivariable logistic regression models to estimate the odds ratio (OR) and the 95% confidence interval (CI) for each index of incense exposure. To select covariates for a base model, we used a forward stepwise method to identify potential confounding factors associated with lung cancer with *p* < 0.05, specifically, age, place of birth, education level, any cancer in first-degree relatives, intake of meat, alcohol consumption, and smoking pack-years. We also included SHS in the base model, although it was not a significant predictor of lung cancer. We removed smoking pack-years from the base model when the analysis was restricted to the never-smokers, and we added years of smoking cessation to the base model when analyses were restricted to ever-smokers.

We formally assessed multiplicative-scale interaction (sometimes referred to as OR modification) between smoking and incense exposure by performing likelihood ratio tests comparing models with and without a product term between smoking (ever, never) and incense exposure (any, none). We evaluated additive-scale interactions (sometimes referred to as risk difference modification) of incense exposure and smoking on lung cancer using the synergy index (SI) (Greenland and Rothman 1998), as follows:


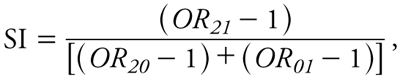
[1]

where *OR_21_* compares lung cancer in high incense users (≥ 2 times/day or ≥ 60 day-years) who smoked with lung cancer in never-users who were never-smokers, *OR_20_* compares high incense users who were never-smokers with never-users who were never-smokers, and *OR_01_* compares never-users who smoked with the same jointly unexposed referent group. Note that subtracting 1 from each OR provides an estimate of the excess relative risk of lung cancer in each group above that in the common referent group of nonusers who were never-smokers (where *OR_00_* = 1). In addition, because incense use was classified into three categories (high, low, and never use), we calculated a second SI comparing joint effects of low incense use and smoking (based on *OR_11_*, *OR_10_*, and *OR_01_* for low use and smoking, low use and never-smokers, and never use and smoking compared with never use and never-smokers). We used a bootstrapping method (Knol et al. 2007) to estimate 95% CIs for the SI and considered additive-scale interactions to be present if the 95% CI excluded one (Yu and Tse 2006b). The same approach was applied to examine additive-scale interactions between incense exposure and residential radon among smokers. We explored exposure–response relationships with gradients of exposure to incense smoke (i.e., as an ordinal categorical variable coded using integer values of 0, 1, 2) by trend tests at an alpha level of 0.05.

## Results

The average age of cases and referents at interview was 66 years (Table 1). More lung cancer cases than referents were smokers and had smoked heavily (≥ 20 pack-years). Compared with referents, cases were more likely to have exposures to SHS and higher living density (fewer square meters per head), and were exposed to higher levels (more cases in the category of the fourth quartile) of radon in their residences. Less than half of the cases and referents had ever cooked at home, and gas was the most frequently used fuel in both groups. Except for cigarette smoking and exposures to SHS and residential radon, no significant differences were observed between the cases and referents in other characteristics (Table 1).

**Table 1:** Selected characteristics of 1,208 lung cancer cases and 1,069 community referents among Hong Kong Chinese men, 2004–2006.^*a*^

Characteristic	Referents [*n* (%)]	Cases [*n* (%)]	*p-*Value
Age at interview*b*		66.2 ± 9.9		65.8 ± 9.5		0.326
Amount of cigarette smoking (pack-years)*b*		17.1 ± 29.0		44.1 ± 33.8		< 0.001
Never		536 (51.7)		132 (11.1)		< 0.001
< 20		172 (16.6)		122 (10.2)		
≥ 20		329 (31.7)		940 (78.7)		
Missing		32		14		
Exposed to environmental tobacco smoke						
Never		281 (26.3)		230 (19.1)		< 0.001
Ever		788 (73.7)		977 (80.9)		
Missing		0		1		
Residential radon exposure (in quartiles)*c*						
First		263 (25.0)		230 (20.3)		0.002
Second		261 (24.8)		302 (26.6)		
Third		267 (25.2)		255 (22.4)		
Fourth		263 (25.0)		349 (30.7)		
Missing		15		72		
Exposed to mosquito coil burning						
Never		967 (90.5)		1066 (88.2)		0.088
Ever		102 (9.5)		142 (11.8)		
Living density (square meters per head)*b*		9.66 ± 10		8.97 ± 7.54		0.068
Residential ventilation						
Poor		530 (49.6)		616 (51.0)		0.501
Good		539 (50.4)		592 (49.0)		
Cook at home						
Never		587 (55.8)		699 (58.4)		0.214
Ever		465 (44.2)		498 (41.6)		
Missing		17		11		
Separate room for cooking at home						
Never		629 (58.9)		743 (61.6)		0.350
Not always		216 (20.2)		237 (19.7)		
Always		223 (20.9)		226 (18.7)		
Missing		1		2		
Use exhaust appliances for cooking at home						
Never		667 (63.0)		791 (66.2)		0.272
Not always		193 (18.2)		195 (16.3)		
Always		199 (18.8)		209 (17.5)		
Missing		10		13		
Types of fuel use						
Never and/or electricity		604 (57.5)		706 (59.0)		0.459
Gas only		359 (34.2)		376 (31.4)		
Liquid and/or gas		63 (6.0)		85 (7.1)		
Solid and/or gas		25 (2.3)		30 (2.5)		
Missing		18		11		
Histology						
Squamous cell carcinoma				272 (22.5)		
Adenocarcinoma				440 (36.4)		
Small-cell carcinoma				118 (9.8)		
Large-cell carcinoma				25 (2.1)		
Other or not otherwise specified				353 (29.2)		
**a**Observations with missing data were not included when calculating the percentage. **b**Mean ± SD. **c**Using the quartile score of community referents as the cut point (first, second, third, and fourth quartile: (< 7.78, 7.78 to < 8.86, ≥ 8.86 to < 9.62, and ≥ 9.62, respectively) to classify different levels of radon exposure.						

The variable with the highest proportion of missing data was radon (72 cases and 15 referents). There were no missing data for smoking status, and few observations with missing data for incense burning (four cases) and SHS (one case) exposure. Data from 1,109 lung cancer cases and 1,015 referents with complete information were included in the analyses. Generally, there were no significant differences in major characteristics (e.g., education, place of birth, alcohol drinking and smoking habits, family cancer history, and meat intakes) between cases or referents with missing data and those included in the analysis, with the exception of age. The referents with missing data were 4.4 years younger, on average, than were referents included in the analyses (data not shown). We examined the correlations among cigarette smoking, incense exposure, and residential radon exposure and found low correlations (correlation coefficients ranged from –0.007 to 0.105) between them.

More cases than referents ever had exposures to incense smoke at home (around 65% and 55%, respectively) (Table 2). The mean ± SD of the duration of exposure to incense burning was 50.3 ± 22.4 years for lung cancer cases and 49.1 ± 21.4 years for the referents; this finding indicates that burning incense was a long-term practice in the homes for most Hong Kong Chinese. In the population as a whole, lung cancer risk was significantly increased among men exposed to frequent incense burning (≥ 2 times/day; OR = 1.26; 95% CI: 1.01, 1.58) and high cumulative incense exposure (≥ 60 day-years; OR = 1.38; 95% CI: 1.10, 1.75) compared with men who were never exposed to incense (Table 2). Incense use did not appear to be associated with lung cancer among never-smokers (e.g., OR = 0.95; 95% CI: 0.59, 1.53 for frequent use vs. never use), but there was some indication that frequent use and high cumulative use (vs. never use) was associated with lung cancer among smokers (trend test *p*-values of 0.039 and 0.007, respectively.) In addition, the synergy index for smoking and frequent incense use (SI = 1.50; 95% CI: 0.98, 2.51) or high cumulative exposure and cigarette smoking (SI = 1.59; 95% CI: 1.06, 2.62) indicated that the joint effect of these two exposures was > 50% than expected assuming additive effects on lung cancer risk. The synergy index for smoking and low level of incense exposure (i.e., < 2 times/day or < 60 day-years) was close to 1.0, and there was no statistical significance.

**Table 2:** Associations between lung cancer risk and exposures to incense burning in the homes of Hong Kong Chinese men, 2004–2006.^*a,b*^

All subjects					Never-smokers					Ever-smokers				
Referents (*n* = 1,069)	Cases (*n* =1,208)	Referents (*n* = 536)	Cases (*n* = 132)	Referents (*n* = 533)	Cases (*n* = 1,076)
Synergy index*c*	*n *(%)	*n *(%)	OR (95% CI)	*n *(%)	*n *(%)	OR (95% CI)	*n *(%)	*n *(%)	OR (95% CI)
Frequency of incense burning (times/day)																				
Never				479 (44.8)		420 (34.8)		1.00		259 (48.3)		61 (46.2)		1.00		220 (41.3)		359 (33.4)		3.38 (2.29, 5.01)
< 2				248 (23.2)		313 (25.9)		1.16 (0.90, 1.48)		111 (20.7)		33 (25.0)		1.15 (0.69, 1.90)		137 (25.7)		280 (26.0)		3.72 (2.46, 5.61)
≥ 2				342 (32.0)		471 (39.0)		1.26 (1.01, 1.58)		166 (31.0)		38 (28.8)		0.95 (0.59, 1.53)		176 (33.0)		433 (40.2)		4.49 (3.02, 6.69)*d*
*p*-Value (test for trend)								0.044						0.908						0.039
		1.50 (0.98, 2.51)*d*																		
Cumulative incense exposure (day-years)																				
Never				479 (44.8)		420 (34.8)		1.00		259 (48.3)		61 (46.2)		1.00		220 (41.3)		359 (33.4)		3.42 (2.31, 5.06)
< 60				280 (26.2)		337 (27.9)		1.05 (0.82, 1.34)		123 (22.9)		32 (24.2)		0.98 (0.59, 1.61)		157 (29.5)		305 (28.3)		3.43 (2.29, 5.14)
≥ 60				310 (29.0)		448 (37.1)		1.38 (1.10, 1.75)		154 (28.7)		39 (29.5)		1.10 (0.68, 1.76)		156 (29.3)		409 (38.0)		5.00 (3.34, 7.51)*e*
*p*-Value (test for trend)								0.007						0.739						0.007
		1.59 (1.06, 2.62)*e*																		
**a**Missing data for 27 lung cancer cases and 36 community referents were not included in the analyses. **b**Models were adjusted for age, place of birth, education level, any cancer in first-degree relatives, intake of meat, and alcohol drinking, as well as smoking pack-years. **c**Synergy index was calculated to examine whether there was an additive-scale interaction of incense exposure and smoking on lung cancer. **d**The synergy index for smoking and frequent incense use (i.e., 2 times/day or more) only. **e**The synergy index for smoking and higher cumulative incense exposure (i.e., ≥ 60 day-years) only.																				

Compared with men who never used incense and who had low radon exposure, the adjusted OR for men who used incense frequently and who had high radon exposure was 1.50 (95% CI: 1.02, 2.22; 240 cases) (Table 3), and the OR for men with high cumulative exposure and high radon was 1.58 (95% CI: 1.06, 2.36; 222 cases) (Table 4). ORs for joint exposure to high radon and frequent or high cumulative incense use were increased when incense use was classified according to quartiles (Table 3 and 4), but power was limited, and none of the synergy indices were statistically significant (data not shown).

**Table 3:** Associations between lung cancer and frequency of incense smoke exposure stratified by exposure status of residential radon among Chinese smoking men, 2004–2006 [OR (95% CI)].*^a,b^*

Frequency of incense exposure		
Levels of radon exposure	No incense		< 2 times/day	≥2 times/day
Residential radon*c*							
Low		1.00			1.10 (0.71, 1.71)		1.09 (0.72, 1.66)
High		1.00 (0.67, 1.48)			1.06 (0.68, 1.65)		1.50 (1.02, 2.22)
Residential radon exposure*d*							
First quartile		1.00			0.81 (0.44, 1.51)		0.88 (0.46, 1.66)
Second quartile		0.96 (0.55, 1.68)			1.45 (0.76, 2.74)		1.21 (0.68, 2.14)
Third quartile		0.83 (0.46, 1.49)			1.10 (0.57, 2.14)		1.12 (0.65, 1.94)
Fourth quartile		1.12 (0.64, 1.97)			1.01 (0.55, 1.84)		1.98 (1.12, 3.49)
**a**Missing data for 31 lung cancer cases and 35 community referents were not included in the analyses. **b**Variables included in the models were age, place of birth, education level, smoking pack-years, years of smoking cessation, alcohol drinking, any cancer in first-degree relatives, and intake of meat. **c**Using the median score of community referents as the cut point to classify the low (score < 8.86) and high level (score ≥ 8.86) of radon exposure. **d**Using the quartile score of community referents as the cut point (first, second, third, and fourth quartile: < 7.78, 7.78 to < 8.86, ≥ 8.86 to < 9.62, and ≥ 9.62, respectively) to classify different levels of radon exposure.							

**Table 4:** Associations between lung cancer and cumulative incense exposure stratified by exposure status of residential radon among Chinese smoking men, 2004–2006 [OR (95% CI)].*^a,b^*

Cumulative incense exposure		
Levels of radon exposure	No incense	< 60 (day-years)	≥ 60 (day-years)
Residential radon*c*						
Low		1.00		0.98 (0.64, 1.50)		1.23 (0.80, 1.91)
High		0.99 (0.67, 1.48)		1.04 (0.68, 1.58)		1.58 (1.06, 2.36)
Residential radon exposure*d*						
First quartile		1.00		0.63 (0.34, 1.15)		1.27 (0.64, 2.52)
Second quartile		0.96 (0.55, 1.67)		1.48 (0.79, 2.75)		1.17 (0.65, 2.09)
Third quartile		0.83 (0.46, 1.49)		1.09 (0.58, 2.06)		1.14 (0.65, 1.99)
Fourth quartile		1.12 (0.64, 1.95)		0.97 (0.54, 1.75)		2.18 (1.21, 3.92)
**a**Missing data of 31 lung cancer cases and 35 community referents were not included in the analyses. **b**Variables included in the models were age, place of birth, education level, smoking pack-years, years of smoking cessation, alcohol drinking, any cancer in first-degree relatives, and intake of meat. **c**Using the median score of community referents as the cut point to classify the low (score < 8.86) and high level (score ≥ 8.86) of radon exposure. **d**Using the quartile score of community referents as the cut point (first, second, third, and fourth quartile: < 7.78, 7.78 to < 8.86, ≥ 8.86 to < 9.62, and ≥ 9.62, respectively) to classify different levels of radon exposure.						

Separate analyses for major histological types of lung cancer did not show evidence of heterogeneity among subtypes, although power to evaluate differences was limited, particularly for large-cell carcinoma (total of 25 cases).

## Discussion

High cumulative exposure or frequent exposure to incense smoke was significantly associated with lung cancer in Chinese men, whereas associations with lower levels of exposure were weak or absent. In addition, associations appeared to be limited to smokers, which is consistent with a synergistic interaction between cigarette smoking and high cumulative incense exposure at home on lung cancer risk. Although power was limited, our results also suggest that radon exposure might modify the association between incense use and lung cancer among men who smoked.

The earliest study on incense burning was conducted by MacLennan et al. in Singapore in the 1970s (MacLennan et al. 1977), which reported a strong relationship between incense burning and lung cancer, especially for women who burned incense while sleeping (OR = 4.11; *p* < 0.01) (MacLennan et al. 1977). However, their results were not confirmed in two subsequent case–referent studies in Taiwan (Chen et al. 1990; Ger et al. 1993). Chen et al. did not find an association between burning incense and adenocarcinoma (age–sex adjusted OR = 0.99; *p* > 0.05) but observed a slightly increased relative risk of small-cell carcinoma of the lung (age–sex adjusted OR = 1.33; *p* > 0.05) in a study using ophthalmic patients as referents (Chen et al. 1990). Using hospital and neighborhood referents, respectively, Ger et al. (1993) observed positive associations with adenocarcinoma among those exposed to incense burning 1–13 times/week (OR = 1.17; 95% CI; 0.34, 3.99 and OR = 1.38; 95% CI: 0.39, 4.94), but inverse associations (OR = 0.27; 95% CI: 0.13, 0.57 and OR = 0.24; 95% CI: 0.10, 0.60) with higher exposures. They also mentioned in their discussion that burning incense was positively associated with lung cancer in females (*p* > 0.10) but was inversely associated in males (*p* < 0.01). Unfortunately, no relevant data could be obtained in their main results, which prevented us from understanding the detailed relationship. Incense burning was identified as a major source of indoor air pollution in Hong Kong (Koo and Ho 1996) but was not associated with lung cancer among nonsmoking women and was inversely associated with lung cancer in smoking women. Koo and Ho (1996) attributed the apparent protective effect in smokers to healthier diets among smoking women who burned incense compared with smoking women who did not burn incense. No data were provided to support the speculation. Our own data in the current study did not support the hypothesis of healthier diet among incense users, as smokers who burned incense at home actually had poorer diets (consumed fewer fresh vegetables and fruits, but more fried food) than those who did not use incense (data not shown). Diet would be a confounder, as a healthier diet in smokers might have explained the inverse association in the study of Koo and Ho (1996), but in our study a poorer diet among smokers may explain the positive association; the potential confounding effect of diet has been adjusted in our study. More recently (in 2003), in a hospital-based study of lung cancer using respiratory clinic patients as referents (including 46% diagnosed with asthma and 5% with chronic obstructive lung diseases), Chan-Yeung et al. (2003) observed an insignificant positive association with daily exposure to incense smoke (vs. no or < 2 years of exposure) among Hong Kong Chinese women (OR = 1.58; 95% CI: 0.77, 3.26; 71 cases) but no association among men (OR = 0.94; 95% CI: 0.56, 1.56; 100 cases) (Chan-Yeung et al. 2003). On the whole, these five previous studies have suffered from several important methodological limitations, including inappropriate choice of referents, inadequate adjustment for major confounding factors, and small study size. Thus, they have provided very limited evidence on the possible association between incense smoke exposure and lung cancer (Chan-Yeung et al. 2003; Chen et al. 1990; Ger et al. 1993; Koo and Ho 1996; MacLennan et al. 1977).

In a prospective cohort study, Friborg et al. (2008) reported that long-term daily use of incense was associated with squamous cell carcinoma of the entire respiratory tract (OR = 1.8; 95% CI: 1.2, 2.6; 59 cases) (particularly in the upper part) among 61,032 Singapore Chinese of the Hokkien or Cantonese dialect group from 1993 to 2005; however, they found no association with lung cancer overall (OR = 0.9; 95% CI: 0.7, 1.2; 93 lung cancer cases). The independent effect of indoor inhalants including incense burning and the possible joint effect with cigarette smoking was further examined by Tang et al. (2010). By combining two hospital-based case–referent studies conducted from 1996 to 1998 and from 2005 to 2008, Tang et al. (2010) found evidence of a strong interaction between burning incense or mosquito coils and ever smoking on lung cancer risk among Singapore Chinese women, with no evidence of an association between burning incense or mosquito coils and lung cancer among never-smokers (Tang et al. 2010). The primary concern of a hospital-based case–referent study is that including referents with diseases that might also be caused by the exposure of interest will bias associations toward the null. This would be a particular concern when respiratory patients are used as referents but would be less of a concern in other cases, as is the case in the study of Tang et al. (2010) who excluded patients admitted for a diagnosis and treatment of cancer or chronic respiratory disease and used a wide range of conditions, including diseases of skin, bones, joints, and connective tissue, and acute trauma as the referents; nevertheless, the generalization of the results to the general population would still be a concern. We did not observe a significant association between mosquito coil burning and lung cancer among Hong Kong Chinese men.

As proposed by Tang et al. (2010), a chronic inflammatory response in the airways induced by smoking might contribute to an interaction between cigarette smoking and incense exposure (Tang et al. 2010). Chronic inflammation in the lungs may increase the interaction of hydroxyl radicals (arising from incense smoke exposure) with DNA which in turn may increase the likelihood of mutations during the replication of DNA (Schottenfeld and Beebe-Dimmer 2010).

Radon is a confirmed human carcinogen, and an effect on lung cancer has been established based on studies of uranium miners (IARC 1988). Radon inside buildings in Hong Kong is released mainly from concrete materials. A territory-wide indoor radon survey conducted by the Environmental Protection Department showed that radon levels of 5% of the residential buildings in Hong Kong were above the safety guideline level of the World Health Organization (WHO) of 200 Bq/m^3^ (Ho 2010). A pooled analysis of 13 European case–referent studies showed a synergistic interaction between radon in homes and smoking on lung cancer, with an absolute risk of lung cancer by 75 years of age due to radon exposure among cigarette smokers estimated to be 25 times greater than the risk among lifelong never-smokers (Darby et al. 2005). In our study male smokers with the highest exposures to residential radon and incense burning were at greater risk of lung cancer than smokers with high incense exposure but low radon exposure, consistent with a synergistic effect of incense and radon, although power was limited for this analysis. Radioactive radon decay products may effectively attach to aerosols, dusts, and other particles in the air and enters our lungs with breathing (WHO 2009). Radon progeny are then deposited on the cell lining of the airways, where the alpha particles as well as the inhaled toxic aerosols or particles (generated by burning incense or cigarettes) may interact to damage DNA, potentially causing an excess risk of lung cancer. The risk may be further enhanced among cigarette smokers because of chronic inflammatory processes.

Burning incense has been a long tradition for worship in many Chinese families, and three incense sticks are usually burnt during each prayer in their homes. Based on one study, nearly 40% of families did not open windows when worshipping (Lung et al. 2003). People exposed to incense smoke may inhale a complex mixture of PM, gaseous products, and many organic compounds. A major shortcoming of the present study is the lack of direct measurements to characterize indoor inhalants. Although direct measurements were not available, some information is available from previous studies for risk assessment (Ho and Yu 2002; Lung et al. 2003; Wang et al. 2007). Ho and Yu (2002) observed a prolonged high exposure to formaldehyde (> 100 µg/m^3^ for 2–3 hr) when incense was burned in the home 2–3 times a day. Lung et al. reported that PM_10_ concentrations near the altar during incense burning were > 9 and 1.6 times the background levels under unventilated and ventilated conditions, with measured concentrations of 723 and 178 μg/m^3^, respectively (Lung et al. 2003). Persistently high levels of PM_10_ and particulate PAHs were detected for at least 6 hr after incense was burned in an unventilated home but were substantially reduced with improved ventilation (Lung et al. 2003). A recent study reported significantly increased DNA damage and decreased DNA repair capacity among temple workers with daily exposure to incense smoke compared with workers in an occupational setting unrelated to benzene, butadiene, or PAH exposure (Navasumrit et al. 2008), which provides supportive evidence that exposure to incense smoke may increase the risk of lung cancer.

The association between incense burning and lung cancer among women may be stronger than in men, because women in traditional Chinese families spend more time in the home. Two previous studies looked at men and women separately and found that the lung cancer risk was positively associated with burning incense for women only, although power was very limited (Chan-Yeung et al. 2003; Ger et al. 1993). We did not find an association between incense smoke exposure and lung cancer among never-smoking men but observed *a*) a significantly increased OR among smokers who were exposed to incense burning, and *b*) a slightly stronger relationship with greater cumulative incense exposure. All previous studies used the frequency of incense burning as an exposure indicator; none of them examined the association between cumulative exposure to incense smoke and lung cancer risk.

Selection bias is possible, as the cases (96%) and referents (48%) had different participant rates. We chose the community referents matched for the district of residents of the cases, which is likely to have improved comparability relative to use of referents who were not from the same districts. The prevalence of ever smoking of our community referents was 49.9%, which was very close to that of a population-based random sample of 1,506 men 26–70 years of age from the same area (54.4%) (Cheng and Ng 2007). However, data on incense burning in the general population are not available.

Recall bias is usually less of a concern for habitual exposure (which is probably what incense burning would be). Residual confounding from cigarette smoking could be a concern, but it should have been dealt with adequately by incorporating the amount and years of smoking as well as years since smoking cessation into the regression models. Given that incense burning is a traditional ritual, it seems likely that men from families who burn incense may differ from families that do not burn incense in many ways, including religious practices that might be relevant to health behaviors, ethnicity, diet, etc. Residual confounding from religious practices could not be ruled out completely, and we acknowledge it as a potential limitation of the study. People burning incense at home may be more likely to worship in temples; however, information on incense smoke exposure outside the home was not collected, which is another limitation of our study.

In conclusion, compared with men who were never exposed to incense in the home, we observed a modest excess risk of lung cancer among men who had relative high exposure to incense smoke in the home, which was restricted primarily to smokers. We also found preliminary evidence that radon exposure might increase risk among smokers with high incense exposure, but power was limited. Our study suggests that exposure to incense smoke in the home may increase the risk of lung cancer among smokers and that exposure to radon may further increase risk. Our study strongly suggests that efforts to prevent lung cancer in the community should include the reduction or minimization of exposures to indoor air pollutants, as well as smoking cessation.
